# One-year unplanned readmission after percutaneous coronary intervention in ST-elevation myocardial infarction: rates, causes, and predictors—a retrospective cohort study

**DOI:** 10.3389/fcvm.2025.1581371

**Published:** 2025-09-16

**Authors:** O. Alkhalaila, A. Rahhal, M. Altermanini, M. S. Abdelghani, M. Shehadeh, K. Shunnar, M. B. Habib, Y. Hailan, M. Barakat, M. H. Alkhateeb, M. Al-Hijji, A. R. Arabi

**Affiliations:** ^1^ Cardiology Department, Heart Hospital, Hamad Medical Corporation, Doha, Qatar; ^2^ Pharmacy Department, Heart Hospital, Hamad Medical Corporation, Doha, Qatar; ^3^ Internal Medicine Department, Hamad General Hospital, Hamad Medical Corporation, Doha, Qatar

**Keywords:** ST-elevation myocardial infarction, readmission, percutaneous coronary intervention, predictors, causes

## Abstract

**Background:**

Unplanned readmissions after percutaneous coronary intervention (PCI) in patients with ST-elevation myocardial infarction (STEMI) significantly impact healthcare systems. However, most of the existing literature focuses on short-term readmission rates and causes, with limited data on long-term readmissions. To date, no studies have evaluated the unplanned readmission post-PCI in STEMI patients within the Arab Gulf region. This study aimed to determine the rates, causes, and predictors of readmission post-PCI among STEMI patients over a one-year follow-up in Qatar, one of the Arab Gulf countries.

**Methods:**

We conducted a single-center retrospective cohort study at Hamad Medical Corporation in Qatar, involving 1,257 patients who underwent PCI during their index STEMI admission between January 1, 2016, and September 30, 2018. Patients were divided into two groups; (1) those who had one or more unplanned readmission within one year after PCI; (2) and those who did not have readmissions. The outcomes evaluated were the rates, causes, and predictors of all-cause and cardiac readmissions within one year post-PCI.

**Results:**

The mean age of the study population was 51 ± 10 years, and male gender presented 96%. The rate of all-cause readmission within one year post-PCI was 11.5%, with 8.2% due to cardiac reasons. Positive predictors of all-cause readmission included female gender (aOR = 4.14, 95% CI 2.10–8.18, *p* < 0.001), chronic kidney disease (aOR = 2.76, 95% CI 1.07–7.08, *p* = 0.035), more than one stent during PCI (aOR = 1.66, 95% CI 1.09–2.55, *p* = 0.019), and clinical heart failure during the index admission (aOR = 2.36, 95% CI 1.49–3.74, *p* < 0.001).

**Conclusion:**

This study highlights the need for targeted management strategies for high-risk populations to reduce readmission rates.

## 1 Introduction

ST-elevation myocardial infarction (STEMI) is a critical cardiac event that requires prompt and effective intervention to reduce mortality and morbidity. Primary percutaneous coronary intervention (PCI) is the standard of care for STEMI, serving as the primary revascularization modality that significantly improves both short- and long-term outcomes. Advances in stent technology, pharmacotherapy, and interventional practices have further enhanced procedural outcomes post-PCI, resulting in reduced in-hospital mortality and complication rates (1, 2). However, unplanned readmissions following PCI in STEMI patients have a substantial impact on patient quality of life and the healthcare system, particularly since some of these readmissions could be preventable by recognizing and addressing modifiable causes and risk factors (3–6).

The nature and impact of readmissions post-PCI, in the context of acute coronary syndrome (ACS) in general and STEMI in particular, are complex. Despite the numerous efforts to reduce these readmissions, their incidence remains substantial, with reported rates roughly ranging between 5% and 20% (3, 7, 8). Most previous studies have primarily focused on short-term readmissions within 30 days (3, 9–20), with limited literature evaluating long-term readmission rates within one year and their predictors post-PCI (7, 21). Moreover, to the best of our knowledge, no study has evaluated unplanned readmissions post-PCI in patients with STEMI in the Arab Gulf region, including Qatar. In this region, the population and cardiac patients tend to be younger compared to other countries, making them a unique group that may experience different post-PCI outcomes.

Previous studies showed that heart failure, recurrent ischemia, and chest pain were the most frequent causes for readmission post-PCI (3, 7, 14, 16). Reported risk factors associated with higher readmission rates include female sex, chronic kidney disease (CKD), and prior heart failure (3, 4, 9, 10).

This study aimed to evaluate the incidence, predictors, and causes of unplanned hospital readmissions within one year following PCI in patients with STEMI. The study will provide valuable insights for healthcare providers to improve treatment and discharge policies and develop outpatient follow-up schedules tailored to high-risk patients, potentially reducing morbidity and mortality following PCI in STEMI patients. Additionally, identifying and addressing the preventable causes of readmissions may help decrease unnecessary costs and optimize bed capacity and financial resources within the healthcare system (22, 23).

## 2 Methods

### 2.1 Study design and population

We conducted a retrospective observational cohort study at Heart Hospital, which is the main tertiary cardiology center with Hamad Medical Corporation in Qatar. The study was approved by Hamad Medical Corporation Medical Research Centre and Institutional Review Board in Qatar (MRC-01-21-474).

The study included all patients admitted to Heart Hospital with a diagnosis of STEMI who underwent PCI during the index admission and were discharged alive between January 1, 2016, and September 30, 2018, using whole population sampling. Patients who died during the index admission or were admitted for elective PCI were excluded. Patients were divided into two groups: those who had one or more unplanned readmissions within one year after PCI (group 1), and those who did not (group 2).

### 2.2 Data collection

Data were extracted from electronic medical records using pretested data collection through MS excel, and data entry was verified by two study authors. Data included baseline demographics, clinical characteristics, comorbidities, initial and peak cardiac biomarkers (troponin-T), echocardiographic parameters, procedural details, inpatient outcomes, discharge medications, and one-year clinical outcomes. The outcomes of interest were the rates, causes, and predictors of all-cause, cardiac readmissions, and heart failure readmission within one year post-PCI.

### 2.3 Statistical analysis

Descriptive statistics were used to summarize baseline characteristics and outcomes. Continuous variables were expressed as mean ± standard deviation or median [interquartile range] where appropriate. Categorical variables were expressed as frequencies and percentages. Univariate logistic regression analysis for baseline patient- and disease-related characteristics was conducted. Then multivariate logistic regression model using pre-specified clinically significant variables was used to identify predictors of readmission, and results were reported as adjusted odds ratios (aOR) with 95% confidence intervals (CI). A *p*-value < 0.05 was considered statistically significant.

## 3 Results

### 3.1 Baseline characteristics

A total of 1,257 patients were included in the analysis (Table 1). The mean age was 51 ± 10 years, and the majority were male (95.9%). Most patients were of Asian origin (76.6%), followed by those from the Middle East (19.9%). A significant proportion were current smokers (50.8%). Common comorbidities included diabetes mellitus (41.8%), hypertension (32.7%), and dyslipidemia (31.0%). Primary PCI was performed in 99.6% of patients, with the most frequent culprit vessel being the Left Anterior Descending artery (LAD) (56.3%), followed by right coronary artery (RCA) (33.3%). Around half of the study population (52.2%) had multivessel disease and 8.1% underwent staged PCI as inpatient, while 14.9% underwent staged PCI as outpatient. Around 17.7% of patients had reduced ejection fraction of less than 40% during the index admission.

**Table 1:** Baseline characteristics of patients admitted with STEMI (*N* = 1,257).

Characteristic	*n* (%)
Age (years)	51 ± 10
Gender	
Male	1,205 (95.9)
Female	52 (4.1)
Region of origin	
Asia	963 (76.6)
Middle East	250 (19.9)
Africa	29 (2.3)
Europe	9 (0.7)
North America	6 (0.5)
Weight (Kg)	76 ± 15
Smoking status	
Smoker	638 (50.8)
Never	396 (31.5)
Ex-smoker	98 (7.8)
Unknown	125 (9.9)
Alcohol use	100 (8.0)
Family history of CVD	
Yes	164 (13.0)
No	583 (46.4)
Unknown	510 (40.6)
Medical History	
Hypertension	411 (32.7)
Diabetes Mellitus	526 (41.8)
Dyslipidemia	389 (31.0)
Heart failure	7 (0.5)
Coronary artery disease	110 (8.7)
PCI	68 (5.4)
Atrial fibrillation	6 (0.5)
Cerebrovascular accident	14 (1.2)
Peripheral artery disease	2 (0.2)
Gastrointestinal bleeding	5 (0.4)
Chronic kidney disease	32 (2.5)
Hypothyroidism	14 (1.2)
Hyperthyroidism	3 (0.2)
Anemia	50 (4.0)
Number of comorbidities	
0	367 (29.2)
1	432 (34.4)
2	267 (21.2)
3	111 (8.8)
4	50 (4.0)
5	22 (1.8)
>5	8 (0.6)
Initial troponin-T	
Mean	933 ± 3,299
Median	52 [244]
25th percentile	18
50th percentile	52
75th percentile	262
90th percentile	1,851
95th percentile	4,820
Peak troponin-T	
Mean	9,487 ± 12,400
Median	6,823 [8,975]
25th percentile	3,268
50th percentile	6,823
75th percentile	12,243
90th percentile	18,917
95th percentile	25,339
Pro-BNP^a^	118 [702]
LV end-systolic diameter (cm)^a^	3.5 [0.7]
LV end-diastolic diameter (cm)^a^	4.9 [0.7]
Procedure details	
Primary PCI	1,252 (99.6)
Thrombolysis	13 (1.1)
Culprit lesion	
LM	2 (0.2)
LAD	708 (56.3)
RCA	418 (33.3)
LCx	126 (10.0)
Ramus	3 (0.2)
Multivessel disease	656 (52.2)
Coronary angiography access	
Radial	1,017 (80.9)
Femoral	240 (19.1)
Staged inpatient PCI	102 (8.1)
Staged outpatient PCI	187 (14.9)
Number of stents	
0	14 (1.1)
1	1,017 (80.9)
2	189 (15.0)
3	30 (2.4)
>3	7 (0.6)
Stent type^b^	
BMS	461 (37.1)
DES	782 (62.9)
Ejection fraction^c^	
<30%	39 (3.1)
30%–39%	183 (14.6)
40%–49%	624 (49.6)
≥50%	403 (32.1)
New mitral regurgitation	195 (15.5)
Mitral regurgitation severity	
Mild	196 (15.6)
Moderate	9 (0.7)
Severe	1 (0.1)
Mechanical support	
IABP	21 (1.7)
VA-ECMO	2 (0.2)

^a^ Presented as median [interquartile range].

^b^ Data of 1,243 subjects who had stent deployment.

^c^ missing data of 8 subjects.

### 3.2 Inpatient outcomes

During the index admission, clinical heart failure was noted in 13.4% of patients, and 55.1% required ICU admission. The median length of stay was 3 days with interquartile range of 1. Other inpatient complications included cardiogenic shock (4.8%), cardiac arrest (6.7%), and acute kidney injury (6.3%) (Table 2).

**Table 2:** Inpatient outcomes of patients admitted with STEMI (*N* = 1,257).

Outcome	*n* (%)
Clinical heart failure	168 (13.4)
Complete heart block	28 (2.2)
Ischemic stroke	6 (0.5)
Cardiogenic shock	60 (4.8)
Cardiac arrest	84 (6.7)
Acute kidney injury	79 (6.3)
Major bleeding	8 (0.6)
Atrial fibrillation	43 (3.4)
ICU admission	692 (55.1)
Length of stay (days)^a^	3 [1]

^a^ Presented as median [interquartile range].

### 3.3 Discharge medications

At discharge, 100% of patients were prescribed aspirin and P2Y12 inhibitors, with clopidogrel being the most common (94.2%), 99.1% were on statins. Other commonly prescribed medications included beta-blockers (93.3%), ACE inhibitors/ARBs (78.6%) (Table 3).

**Table 3:** Discharge medications of patients admitted with STEMI (*N* = 1,257).

Medication	*n* (%)
Aspirin	1,257 (100)
P2Y12 inhibitor	
Clopidogrel	1,184 (94.2)
Ticagrelor	73 (5.8)
Anticoagulant	
None	1,219 (97.0)
Warfarin	36 (2.9)
Rivaroxaban	2 (0.2)
Beta-Blocker	1,173 (93.3)
Statin	1,246 (99.1)
ACE inhibitor/ARB	988 (78.6)
Proton pump inhibitor	504 (40.1)
Loop diuretic	165 (13.1)
Polypharmacy	906 (72.1)

### 3.4 One-year clinical outcomes

The all-cause readmission rate within one year was 11.5%, with 8.2% attributed to cardiac causes and 3.3% to non-cardiac causes. The most common cardiac causes for readmissions were heart failure (35.9% of cardiac causes) and unstable angina (33.0%), followed by NSTEMI (15.5%) and STEMI (4.9%). Nonspecific chest pain accounted for 6.8% of cases. The median time to readmission was 58 days (Table 4).

**Table 4:** 1-year clinical outcomes of patients admitted with STEMI (*N* = 1,257).

Outcome	*n* (%)
Readmission	145 (11.5)
Readmission for cardiac reason	103 (8.2)
Readmission for non-cardiac reason	41 (3.3)
Reason for cardiac admission^a^	
STEMI	5 (4.9)
NSTEMI	16 (15.5)
Unstable angina	34 (33.0)
Stent thrombosis	2 (1.9)
Heart failure	37 (35.9)
Nonspecific chest pain	7 (6.8)
Arrhythmia	1 (1)
Conduction disorder	1 (1)
Time to readmission (days)	58 [169]

^a^ Data for the 103 subjects who were reemitted for a cardiac reason.

### 3.5 Predictors of all-cause readmission

Multivariate analysis identified female gender (aOR = 4.14, 95% CI 2.10–8.18, *p* < 0.001), chronic kidney disease (CKD) (aOR = 2.76, 95% CI 1.07–7.08, *p* = 0.035), use of more than one stent during PCI (aOR = 1.66, 95% CI 1.09–2.55, *p* = 0.019), and clinical heart failure during the index admission (aOR = 2.36, 95% CI 1.49–3.74, *p* < 0.001) as significant predictors of all-cause readmission (Table 5).

**Table 5:** Predictors of all-cause readmission post STEMI (*N* = 1,257).^a^

Variable	aOR (95% CI)	*P*-value
Female gender	4.14 (2.10–8.18)	<0.001
Family history of CVD	1.01 (0.59–1.72)	0.975
Hypertension	0.96 (0.64–1.46)	0.858
Coronary artery disease	1.60 (0.84–3.05)	0.151
Chronic kidney disease	2.76 (1.07–7.08)	0.035
Diabetes mellitus	0.94 (0.63–1.43)	0.785
>3 comorbidities	0.96 (0.42–2.22)	0.928
Multivessel coronary artery disease	1.33 (0.90–1.95)	0.153
>1 stent	1.66 (1.09–2.55)	0.019
Mitral regurgitation	0.92 (0.57–1.51)	0.750
Clinical heart failure	2.36 (1.49–3.74)	<0.001
ICU admission	1.21 (0.82–1.79)	0.343
Polypharmacy (>5 medications)	1.09 (0.68–1.74)	0.715

Gray shade row indicates statistically significant.

^a^ Adjusted OR > 1 with a significant *p*-value indicates a positive predictor of all-cause readmission post STEMI. CVD, cardiovascular disease; ICU, Intensive Care Unit.

### 3.6 Predictors of cardiac readmission

Predictors of cardiac readmission included female gender (aOR = 3.20, 95% CI 1.46–7.00, *p* = 0.004), CKD (aOR = 3.65, 95% CI 1.32–10.09, *p* = 0.013), and clinical heart failure (aOR = 2.98, 95% CI 1.79–4.96, *p* < 0.001) (Table 6).

**Table 6:** Predictors of cardiac readmission post STEMI (*N* = 1,257).

Variable	aOR (95% CI)	*P*-value
Female gender	3.20 (1.46–7.00)	0.004
Family history of CVD	1.29 (0.66–2.52)	0.453
Hypertension	0.90 (0.55–1.47)	0.681
Coronary artery disease	1.91 (0.94–3.86)	0.072
Chronic kidney disease	3.65 (1.32–10.09)	0.013
Diabetes mellitus	0.98 (0.61–1.58)	0.944
>3 comorbidities	0.74 (0.28–1.91)	0.528
Multivessel coronary artery disease	1.30 (0.82–2.04)	0.262
>1 stent	1.45 (0.88–2.38)	0.144
Mitral regurgitation	1.22 (0.72–2.10)	0.466
Clinical heart failure	2.98 (1.79–4.96)	<0.001
ICU admission	1.12 (0.71–1.78)	0.623
Polypharmacy (>5 medications)	1.12 (0.64–1.95)	0.696

Gray shade row indicates statistically significant.

CVD, cardiovascular disease; ICU, Intensive Care Unit.

### 3.7 Predictors of readmission for heart failure

Female gender (aOR = 3.80, 95% CI 1.22–11.86, *p* = 0.021), CKD (aOR = 4.56, 95% CI 1.22–17.03, *p* = 0.024), mitral regurgitation (aOR = 3.13, 95% CI 1.39–7.03, *p* = 0.006), and clinical heart failure (aOR = 4.82, 95% CI 1.53–15.15, *p* = 0.007) were associated with an increased likelihood of readmission due to heart failure (Table 7).

**Table 7:** Predictors of readmission due to heart failure post STEMI (*N* = 1,257).

Variable	aOR (95% CI)	*P*-value
Female gender	3.80 (1.22–11.86)	0.021
Hypertension	1.10 (0.46–2.60)	0.835
Chronic kidney disease	4.56 (1.22–17.03)	0.024
Diabetes mellitus	1.09 (0.48–2.49)	0.841
>3 comorbidities	1.56 (0.50–4.86)	0.445
Mitral regurgitation	3.13 (1.39–7.03)	0.006
Clinical heart failure	4.82 (1.53–15.15)	0.007
ICU admission	1.34 (0.52–3.42)	0.542
EF < 30%	1.97 (0.69–5.62)	0.204
Loop diuretics use	2.04 (0.66–6.33)	0.217

Gray shade row indicates statistically significant.

EF, ejection fraction; ICU, Intensive Care Unit.

## 4 Discussion

Unplanned readmissions following PCI in patients with STEMI impact patient quality of life and place considerable strain on the healthcare system. They may also be considered as indicators of care quality, making it essential to evaluate their causes and predictors (3–6).

Our study provides comprehensive data on the rates, causes, and predictors of unplanned readmissions within one-year post-PCI in patients with STEMI. The overall unplanned one-year readmission rate was 11.5%, with 8.2% of the readmissions being cardiac-related and 3.3% due to non-cardiac causes. These results are consistent with the broader literature. However, compared to our study, most of the related studies evaluated a relatively short-term readmission within 30 days (8, 9, 11–13, 15, 17). In a multicenter randomized trial involving 1,137 STEMI patients undergoing PCI, the rate of all-cause re-hospitalization, including both cardiac and non-cardiac causes, at one year was 18.6%, which is slightly higher than the incidence observed in our study (7). Kwok et al. reported that 30-day readmission rates range between 4.7% and 15.6% in patients undergoing PCI, aligning with our findings over a longer one-year follow-up (3). Additionally, Rymer et al. and Jang et al. further support our results with similar 30-day readmission rates after PCI, reported as 14% and 12%, respectively, indicating that the majority of readmissions occur early, within the first month post-PCI (8, 15).

The variance between studies in the timeframe of follow-up (30-day vs. one-year) is critical in understanding and comparing readmission rates. Despite these variations, our study's readmission rate falls within the expected range for this patient population. The median time to readmission in our study was 58 days, which means that more than half of the patients were readmitted within the first two months after being discharged. This suggests that the risk of readmission is highest early post-discharge with the incidence declining thereafter over the one-year follow-up period.

In our cohort, among the cardiac readmissions, the most common causes were heart failure (35.9%) and unstable angina (33.0%), followed by NSTEMI (15.5%) and STEMI (4.9%). Nonspecific chest pain accounted for 6.8% of cases. Likewise Sud et al., found that the leading causes of 30-day readmission after STEMI were congestive heart failure (25.7%) and ACS (9.4%) (14). Similarly, Tripathi et al. also identified heart failure and ischemic heart disease as the most common etiologies for readmission (16). Spitzer et al. reported that one-year readmissions post-PCI in STEMI patients due to chest pain without evidence of ischemia were more frequent than those with ischemic evidence (20.4% vs. 16.9%, respectively). These observations align with our findings, where unstable angina was a more common cause of readmission compared to STEMI and NSTEMI combined. This highlights the prevalence of non-ischemic chest pain as a significant contributor to readmissions in this patient population (7).

In our multivariate analysis, we identified **female gender**, **CKD**, and **clinical heart failure** during the index admission as significant predictors of all-cause readmissions, cardiac-related readmissions, and readmissions due to heart failure. Additionally, **the use of more than one stent during PCI** was found to be a predictor of all-cause readmission, while **mitral regurgitation** was a significant predictor of heart failure-related readmission.

Interestingly, our study did not find age or reduced LVEF to be independent predictors, contrary to prior studies (3, 7, 24). These differences may reflect our unique study population characteristics. As noted in the Introduction, the majority of patients were younger males (mean age 51 years; 95.9% male), and the prevalence of certain comorbidities such as peripheral vascular disease was low. Additionally, our cohort was predominantly composed of migrant workers from South and Southeast Asia, who may have different health behaviors, access patterns, and outcomes compared to Western populations.

Kwok et al. also identified female sex, CKD, and heart failure as major predictors of readmission, closely matching our results (3). Similarly, Arnold *et al*. found that female sex and prior heart failure were strong predictors of re-hospitalization for ACS post-PCI, highlighting the commonality of these risk factors across different populations (4).

Notably, O'Brien et al. observed that women, particularly younger women, had significantly higher risks of readmission after STEMI compared to men (9). Similarly, studies by Atti *et al*., Steitieh et al., and Dreyer RP et al., also documented higher readmission rates among women (10, 11, 25). These findings align with our study, where female sex was associated with a 4-fold increase in the likelihood of readmission reinforcing the evidence that gender plays a critical role in post-STEMI outcomes.

While our study focused on readmissions over one-year duration post-PCI, many of the referenced studies primarily assessed 30-day readmission (3, 4, 8). Despite this difference, the predictors of readmission remain similar across both short and long-term follow-up, indicating that certain factors (e.g., female gender, CKD, heart failure) consistently influence readmission risk over time. However, our study did not identify some predictors noted in other studies, such as LVEF, age, diabetes, and peripheral vascular disease (3, 7, 24). This discrepancy may be attributed to differences in the study populations, healthcare settings, or the relatively low prevalence of these conditions in our cohort.

The strong association between clinical heart failure during the index admission and subsequent readmissions reflects the vulnerability of this subgroup and highlights the importance of optimal management of heart failure in STEMI patients. Heart failure may contribute to a higher readmission risk through multiple mechanisms; including persistent volume overload, residual myocardial dysfunction, arrhythmias, and medication intolerance. These patients usually require intensive monitoring, strict adherence to multi medications, and close follow-up to avoid decompensation. Suboptimal outpatient care or lack of structured heart failure programs may further increase their risk of readmission.

By understanding the factors driving post-PCI readmissions in STEMI patients, valuable insights can be gained that may help improve treatment protocols, discharge planning, and outpatient follow-up schedules. Tailoring these aspects to high-risk patients could potentially reduce morbidity and mortality rates. Additionally, addressing preventable causes of readmission can help lower unnecessary costs, conserve bed capacity, and optimize financial resources within the healthcare system (22, 23).

This study was a retrospective cohort study with inherit limitations. First, participants’ completion of the one year follow up duration was not ensured in view of the retrospective design, which might have under-estimated the readmission rates. Second, we determined the readmission rates using the electronic medical records at Hamad Medical Corporation without accounting for admissions to other health sectors. Nevertheless, Hamad Medical Corporation is the leading healthcare sector in Qatar and the study site is the primary cardiac tertiary center and the main referral center in the country with an integrated electronic system across the organization, which limits the possibility of missing readmission outcomes. Third, the study population was relatively young with a mean age of 51 years which might have contributed to limited impact of comorbidities as predictors of readmission.

The male predominance in our study population (95.9%) reflects the demographic structure of Qatar, where the majority of the population consists of immigrant workers who are predominantly male. According to the World Bank, males account for approximately 71.52% of the total population. This disparity is largely attributed to the influx of male migrant workers, particularly in the construction and service industries*.* Therefore, the gender distribution in our study represents the underlying population rather than any selection or randomization bias.

Based on our findings, future research should focus on developing and validating predictive models tailored to Middle Eastern and South Asian populations, incorporating demographic, procedural, and social factors. Prospective cohort studies or multicenter registries could assess the generalizability of our results and help refine patient-specific post-discharge interventions. In addition, qualitative studies exploring psychosocial determinants of readmission—especially among women and younger patients—may uncover modifiable factors. Finally, randomized trials evaluating structured discharge planning and heart failure transitional care in high-risk STEMI patients could provide evidence to reduce preventable readmissions.

## 5 Conclusion

The rate of one-year unplanned readmissions after PCI among patients with STEMI was 11.5%, with over two-thirds of these readmissions attributed to cardiac causes. Acute coronary syndromes and heart failure were the most common causes of readmission. Identified predictors of all-cause readmission were female gender, CKD, multiple stents, and clinical heart failure. Implementing targeted follow-up strategies and personalized discharge planning for high-risk groups may help reduce unplanned readmissions and optimize resource utilization.

## Data Availability

The raw data supporting the conclusions of this article will be made available by the authors, without undue reservation.
